# Impact of a point-of-care urine tenofovir assay on adherence to HIV pre-exposure prophylaxis among women in Kenya: a randomised pilot trial

**DOI:** 10.1016/S2352-3018(24)00125-5

**Published:** 2024-07-05

**Authors:** Monica Gandhi, David V Glidden, Deepalika Chakravarty, Guohong Wang, Charlene Biwott, Peter Mogere, Gakuo Maina, Irene Njeru, Catherine Kiptinness, Phelix Okello, Matthew A Spinelli, Purba Chatterjee, Jennifer Velloza, Vallery Ogello, Andrew Medina-Marino, Hideaki Okochi, Nelly R Mugo, Kenneth Ngure

**Affiliations:** Division of HIV, Infectious Diseases, and Global Medicine, Department of Medicine (Prof M Gandhi MD, M A Spinelli MD, H Okochi PhD) and Department of Epidemiology and Biostatistics (Prof D V Glidden PhD, P Chatterjee, J Velloza PhD), University of California San Francisco, San Francisco, CA, USA; Division of Prevention Science, Center for AIDS Prevention Studies, University of California San Francisco, San Francisco, CA, USA (D Chakravarty MS); Research and Development, Toxicology Unit, Abbott Laboratories, Claremont, CA, USA (G Wang PhD); Center for Clinical Research, Kenya Medical Research Institute, Thika, Kenya (C Biwott MBCHB, P Mogere BSc, G Maina MSc, I Njeru SSc, C Kiptinness MPH, P Okello BA, V Ogello BA, Prof N R Mugo MBChB, Prof K Ngure PhD); The Desmond Tutu HIV Centre, University of Cape Town, Cape Town, South Africa (Prof A Medina-Marino PhD); Department of Psychiatry, Perelman School of Medicine, University of Pennsylvania, Philadelphia, PA, USA (Prof A Medina-Marino); Department of Global Health, University of Washington, Seattle, WA, USA (Prof N R Mugo); School of Public Health, Jomo Kenyatta University of Agriculture and Technology, Juja, Kenya (Prof K Ngure)

## Abstract

**Background:**

Adherence challenges with oral tenofovir-based pre-exposure prophylaxis (PrEP) are common. We developed a point-of-care assay to objectively assess tenofovir in urine and conducted a pilot trial examining the impact of counselling informed by use of this urine assay on long-term PrEP adherence.

**Methods:**

This randomised trial enrolled women not in serodiscordant partnerships 3 months after PrEP initiation at the Kenya Medical Research Institute to compare standard-of-care adherence counselling versus counselling informed by the urine assay (urine-test counselling group) every 3 months for 12 months. In the standard of care group, urine samples were stored and tested at study end without participant feedback. Here we report the adherence primary outcome of hair concentrations of tenofovir at 12 months as a long-term metric (undetectable levels defined long-term non-adherence), as well as urine concentrations of tenofovir at each visit as a short-term adherence metric and acceptability of the assay assessed by quantitative surveys. Data were analysed by randomisation group. This completed trial was registered with ClinicalTrials.gov (NCT03935464).

**Findings:**

From March 17, 2021 to Jan 18, 2022 we enrolled 49 women in the urine-test counselling group and 51 in the standard of care group; retention was 86 (86%) of 100. Nine (21%) of 42 in the urine-test counselling group had hair samples at 12 months with tenofovir concentrations below the limit of quantification compared with 15 (37%) of 41 in the standard of care group. The relative odds of long-term non-adherence in the standard of care group compared with urine-test counselling were 3·53 (95% CI 1·03–12·03; p=0·044). Pre-intervention, urine tenofovir was detectable in 65% in the urine-test counselling group and 71% in the standard of care group (p=0·68). At 12 months, 31 (72%) of 43 in the intervention group had detectable urine tenofovir compared with 19 (45%) of 42 in the standard of care group (p=0·0015). 40 (93%) of 43 participants liked the test very much and only one disliked the test. One participant in the standard of care group was withdrawn at the 6-month visit due to HIV seroconversion.

**Interpretation:**

A low-cost urine tenofovir assay to inform PrEP counselling resulted in improvement in both short-term and long-term metrics of adherence. This urine tenofovir assay could help to improve long-term PrEP adherence.

**Funding:**

National Institute of Allergy and Infectious Diseases and National Institutes of Health.

## Introduction

Access to oral pre-exposure prophylaxis (PrEP) is being expanded worldwide for the prevention of HIV infection. PrEP with tenofovir disoproxil fumarate plus emtricitabine is provided without fees for PrEP users in many countries in sub-Saharan Africa, including Kenya. However, adherence to oral PrEP has been low, especially among important populations such as sexually active women without serodiscordant partners (where the partner is not known to be living with HIV),^1,2^ including adolescent girls and young women. In sub-Saharan Africa, adolescent girls and young women accounted for more than 60% of all new HIV infections in 2021. In Kenya, adolescent girls and young women have an HIV prevalence double that of their male peers (2·6 *vs* 1·3%).^3^ Despite this risk, a systematic review and meta-analysis showed that 41% of participants prescribed PrEP in 20 countries discontinued the agent within 6 months, with higher rates of discontinuation in sub-Saharan Africa.^4^ Importantly, discontinuation rates were lower in studies that included adherence interventions than in those which did not. Even when PrEP was continued, adherence was suboptimal in 38% who remained on the medication.^4^ In a recent analysis of over 20 000 patients on PrEP in the USA, only 44·5% were consistently adherent to PrEP, with higher rates of discontinuation in women.^5^ Clearly, low-cost scalable adherence interventions are needed to optimise outcomes on PrEP.

Interventions to improve PrEP adherence, including two-way SMS text messaging, adherence clubs, motivational interviewing, contingency management, and others, have had mixed success and might not be widely cost-effective or scalable.^6^ One proposed way to intervene upon adherence is to counsel patients on increasing pill-taking on the basis of feedback about their PrEP drug concentrations. However, most pharmacological metrics to measure tenofovir or its metabolite, tenofovir-diphosphate, from oral PrEP require liquid chromatography tandem mass spectrometry (LC-MS/MS). The equipment for LC-MS/MS is expensive, and the laboratory assays require trained personnel and time. The time delay in getting results back on pharmacological metrics of adherence from a reference laboratory to the field can therefore be prolonged. Despite the acceptability of drug detection voiced by participants in PrEP trials,^7^ adherence interventions using drug concentration feedback in the context of PrEP have not been consistently successful when using assays that have prolonged processing times,^8–11^ such as tenofovir concentrations in plasma or tenofovir-diphosphate concentrations in dried blood spots.

Our group at the University of California, San Francisco (UCSF) Hair Analytical Laboratory, in collaboration with Abbott Rapid Diagnostics, has recently developed a low-cost point-of-care urine-based lateral flow immunoassay to objectively, rapidly, and accurately measure short-term adherence to tenofovir-based regimens.^12,13^ The assay has been validated against the gold standard of LC-MS/MS to accurately measure tenofovir ingestion.^12^ This real-time metric allows for biofeedback on drug concentrations at a clinic or research visit to rapidly tailor adherence counselling.

In the context of antiretroviral therapy, the urine tenofovir assay has been shown to increase adherence and subsequent virological suppression rates in sub-Saharan Africa.^14^ In the previous study by our group, participants taking tenofovir, lamivudine, and dolutegravir with viral loads greater than 1000 copies per mL were enrolled from 42 clinics across Namibia.^14^ WHO recommends enhanced adherence counselling for patients without virological suppression and these participants had undergone 3 months of enhanced adherence counselling without success. Of 211 participants who received subsequent enhanced adherence counselling informed by the urine tenofovir assay results, 97% achieved virological suppression by 9 months. A previous study in Namibia (providing counterfactual data) had shown only a 33% virological suppression rate with enhanced adherence counselling alone, suggesting that the urine assay-informed intervention increased virological suppression rates significantly (p<0·001).^14^ The urine tenofovir test was acceptable to both patients and providers, with 88% of participants and 93% of providers in the study in Namibia and other studies wanting to continue to use the test in practice.^14^ Encouraging results of this before-and-after intervention involving a point-of-care adherence metric for antiretroviral therapy will be rigorously tested in a randomised clinical trial.

In the context of PrEP, no previous trial has examined the effect of the urine tenofovir assay on adherence rates to the oral medication. We have shown a strong relationship between tenofovir pill-taking and drug concentrations in urine^15^ and determined the appropriate cutoff of the urine assay to classify 98% of patients who took a dose 24 h ago as adherent, with greater than 99% accuracy for those taking tenofovir daily.^12,16^ We have also demonstrated the predictive potential of low tenofovir adherence by the urine assay on future seroconversion events in PrEP trials, specifically the Partners PrEP study and the Preexposure Prophylaxis Initiative (iPrEx) open-label extension study.^17,18^ Preliminary acceptability and feasibility findings of the urine tenofovir assay in this study determined with qualitative methods have been published previously,^19^ but quantitative acceptability data have not been published and are being reported here. In this manuscript, we aimed to describe the results of the first trial to examine the effect of a targeted counselling intervention informed by the urine tenofovir assay on PrEP adherence rates among women not in serodiscordant relationships in Kenya.

## Methods

### Study design

The Point-of-Care Urine Monitoring for Adherence (PUMA) study was a pilot randomised clinical trial to evaluate the acceptability, feasibility, and effect on PrEP adherence over time of real-time monitoring and feedback via the urine assay among women not in serodiscordant couples.

The study was done at the Kenya Medical Research Institute (KEMRI) Partners in Health and Research Development (PHRD) clinical research centre in Thika, Kenya (a city 42 km from Nairobi). The clinical research centre in Thika is associated with five community sites from which participants are recruited for studies including surrounding voluntary counselling and testing centres, churches, and community organisations which mobilised around women’s voluntary counselling and testing promotion. All study visits were done at the KEMRI PHRD clinical research centre.

The study obtained ethics approval from both the KEMRI Institutional Review Board and the UCSF Institutional Review Board. The protocol has been published previously.^20^ The trial was registered with ClinicalTrials.gov (NCT03935464).

### Participants

Participants were recruited with the following eligibility criteria: cisgender female aged 18 years or older, HIV-negative on HIV rapid test, not currently enrolled in an HIV prevention clinical trial, not currently in a serodiscordant couple, willingness to be randomly assigned to point-of-care tenofovir drug testing, ability and willingness to provide written informed consent, and no demonstrated contraindication to the use of tenofovir disoproxil fumarate. To minimise study attrition, we recruited participants at their clinic visit that occurred 3 months after PrEP initiation. Women who were pregnant or breastfeeding were eligible for the study. All participants provided written informed consent for the study.

### Randomisation and masking

**Figure 1:**
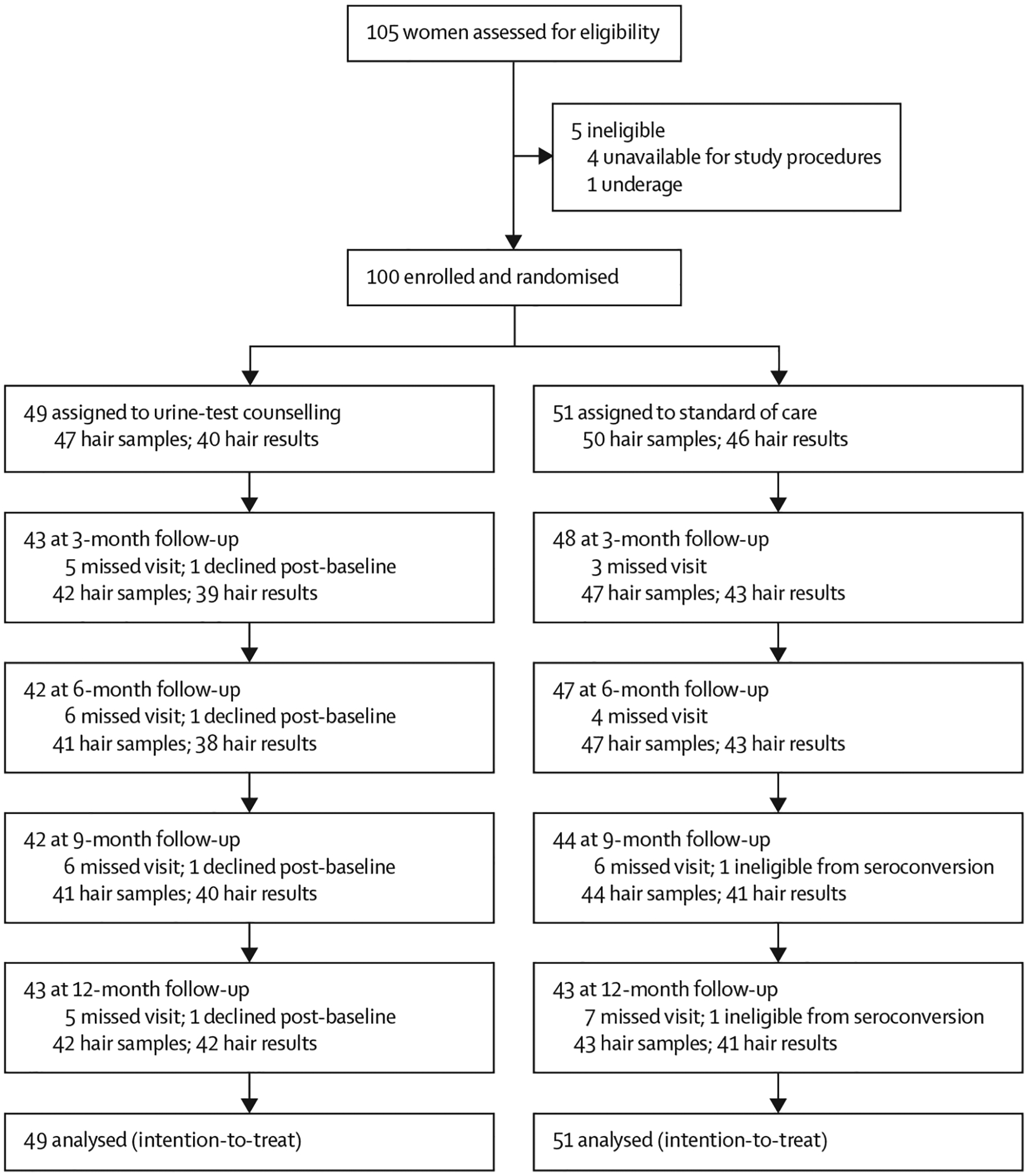
Trial profile Missing hair samples are due to participants declining to provide one; missing hair results are due to inadequate quantity of hair sampled.

After enrolment, participants were randomly assigned (1:1) to the urine-test counselling intervention group or the standard of care group (figure 1). The randomisation scheme used variable-sized (4, 6, 8) blocks, generated using SAS and incorporated into the Research Electronic Data Capture (REDCap)^21^ data collection platform’s randomisation module. A small number of senior staff at the study clinic who were blinded to the randomisation scheme itself were given access to perform the randomisation within REDCap. The study database architect based at UCSF who created the randomisation scheme was the sole person to be unmasked to it. The laboratory team at the UCSF Hair Analytical Laboratory was masked to the assignment group for each participant for whom they were analysing hair samples for tenofovir concentrations.

### Procedures

**Figure 2:**
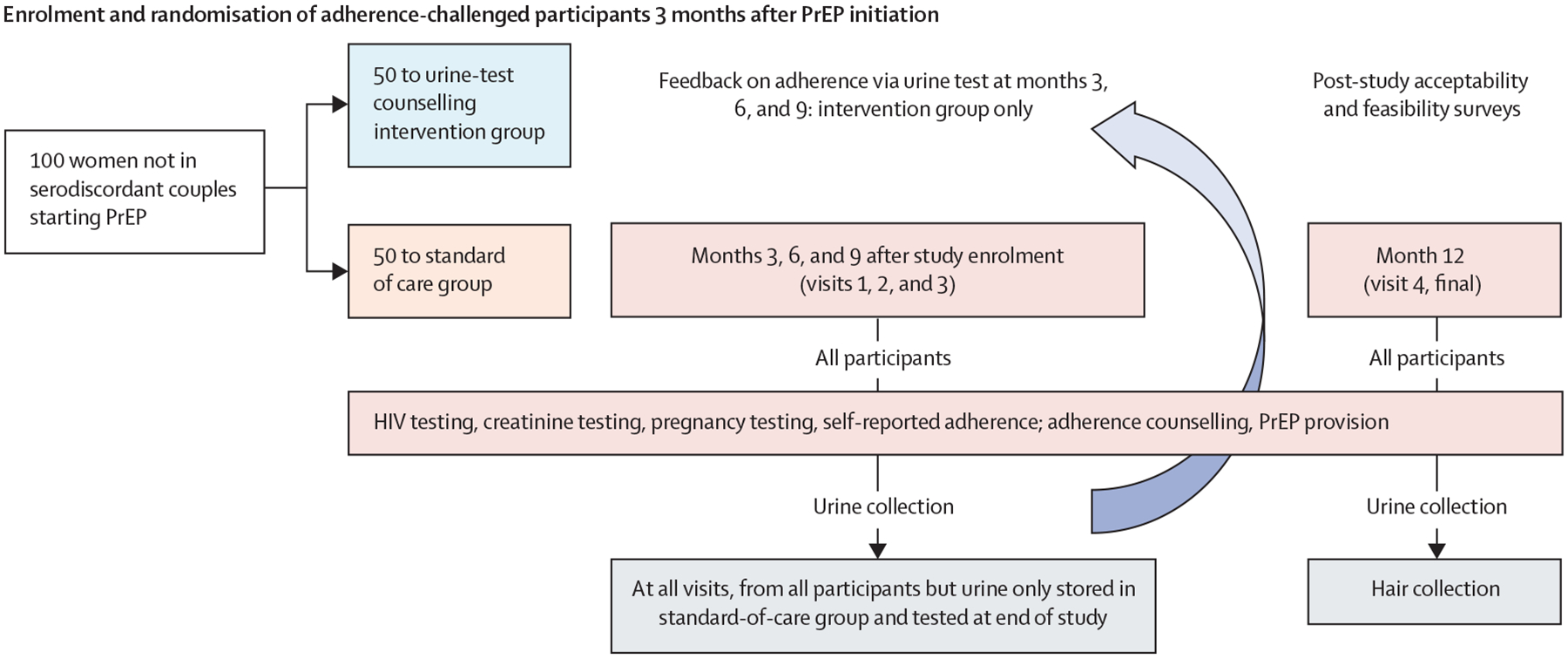
Study design PrEP=pre-exposure prophylaxis.

Upon randomisation, study visits occurred at 0 (baseline visit), 3, 6, 9, and 12 months after study enrolment (figure 2). Under the 2022 Kenya PrEP guidelines, all people initiating PrEP undergo clinical assessment to ask about symptoms of acute HIV infection and receive rapid HIV tests and creatinine testing. People who qualify for and initiate PrEP are subsequently seen in 1 month, 3 months, and then every 3 months for repeat HIV testing, continued risk assessment, and adherence counselling for daily PrEP under the standard of care. Standard adherence counselling involves discussion of the importance of PrEP pill-taking, identifying adherence barriers, and providing recommendations to overcome those barriers. At each study visit for all participants, HIV testing was done with condom promotion and provision, and participants responded to a brief survey.

Urine samples were collected at every visit for participants in both groups but stored for participants in the standard of care group for testing at the end of the study. For participants in the intervention group, the urine samples were immediately tested via the point-of-care assay (appendix p 2) by laboratory staff and results were conveyed to the clinical providers for real-time feedback to participants.^19^ The urine assay takes 2–3 min to provide results. Feedback to the participants on their test results were adapted from the HPTN 082 trial^22^ and delivered in a supportive manner by providers. The counselling messages for this trial (appendix p 3) were first piloted in a small group of women on PrEP at the research clinic for refinement before actual implementation.^19^ Pictures of each urine tenofovir assay done were uploaded into the research database for quality assurance monitoring. Small hair samples (approximately 50–100 strands) were collected at 12-month visits with previously described methods.^23^

### Outcomes

The primary outcomes of the pilot study were feasibility of the intervention assessed as retention at 12 months; acceptability of urine tenofovir testing among women receiving PrEP assessed via quantitative surveys of intervention group participants; and preliminary impact on adherence as assessed by a long-term metric of adherence assessed as impact on hair tenofovir concentrations, a biomarker of long-term adherence^24^ (measuring adherence over the past 8 weeks), at 12 months after randomisation. We also report an analysis of intervention on urine tenofovir test results, a marker of short-term adherence (drug consumption over the past 1–4 days).

The cutoffs for adherence on the lateral flow assay for tenofovir concentrations in urine (1500 ng/mL) and hair (0·002 ng/mg) were established in directly observed therapy studies.^12,24^ The urine assay is coded as “yes” or “no” on the basis of the number of lines to indicate whether tenofovir is present at greater than 1500 ng/mL as previously described.^12^ Tenofovir concentrations in hair samples were measured at the UCSF Hair Analytical Laboratory with validated methods^25^ peer reviewed and approved by the National Institutes of Health Division of AIDS’ Clinical Pharmacology and Quality Assurance Program.^26^

The acceptability outcome was assessed among participants in the urine-test counselling intervention group at 6 months and 12 months as captured by use of three questions with categorical responses: “How did knowing the PUMA result during the study visit impact your PrEP adherence after your visit?”; “How much did you like or dislike receiving PrEP adherence results in real-time?”; and “If you were not part of a study, how likely would you be to want to know PrEP adherence results using PUMA?”.

Safety and adverse events were also assessed. For the purposes of this study, only serious adverse events deemed related to study procedures are reported.

### Statistical analysis

A previous analysis across PrEP studies had examined differences in adherence assessed via tenofovir concentrations in hair.^27^ The sample size of 100 participants was chosen to yield 80% power to detect a difference of 0·68 log (tenofovir hair concentrations) between the groups assuming a standard deviation of 1·2 log tenofovir with a 0·05 level two-sample *t* test.

Study data were collected at the KEMRI PHRD research clinic using the secure REDCap platform hosted at UCSF.^21^ Participants were analysed in accordance with the group to which they were randomly assigned. Tenofovir concentrations below the limit of quantification in hair, specifically less than 0·002 nanograms per milligram, defined long-term non-adherence.^24^ The percent of positive urine tenofovir tests in each group were compared via χ2 tests. The percent of positive urine tenofovir tests and tests with non-detection of tenofovir in hair were modelled by logistic regression with a robust variance to account for clustering over time. We compared hair tenofovir detection stratified by result of the urine tenofovir assay by using the Pearson χ2 test. The predictors in these models were randomisation group, study visit, and their interaction. Responses to the acceptability questions are summarised and reported as proportions. All analyses were done with SAS version 9.4 and Stata version 18.0.

The study had a Data Safety and Monitoring Board composed of a statistician from the Harvard Center for Biostatistics in AIDS Research and two high-level leaders and HIV investigators from KEMRI and the Kenyatta National Hospital in Nairobi, both of whom were uninvolved in the study. The Data Safety and Monitoring Board met semiannually throughout the study and at the end of study enrolment to approve study procedures, as well as safety and effectiveness outcomes.

### Role of the funding source

The funders of the study peer reviewed and approved the study protocol through the Division of AIDS Clinical Science Review Committee. The funders had no role in data collection, data analysis, data interpretation, or writing of the report.

## Results

From March 17, 2021 to Jan 18, 2022, 105 women were approached for the study and 100 were enrolled and randomly assigned: 49 to the urine-test counselling intervention group and 51 to the standard of care counselling group (figure 1). One participant in the standard of care group was withdrawn at the 6-month visit due to HIV seroconversion. Retention in the study was 86% in both groups. All available data were analysed in this intention-to-treat analysis.

At baseline, groups were well matched in terms of age and knowledge of partner’s HIV status (table 1). 45% of participants in the urine-test counselling group and 39% in the standard of care group had engaged in transactional sex in the previous month, and most participants self-reported daily PrEP intake in the previous month.

**Figure 3:**
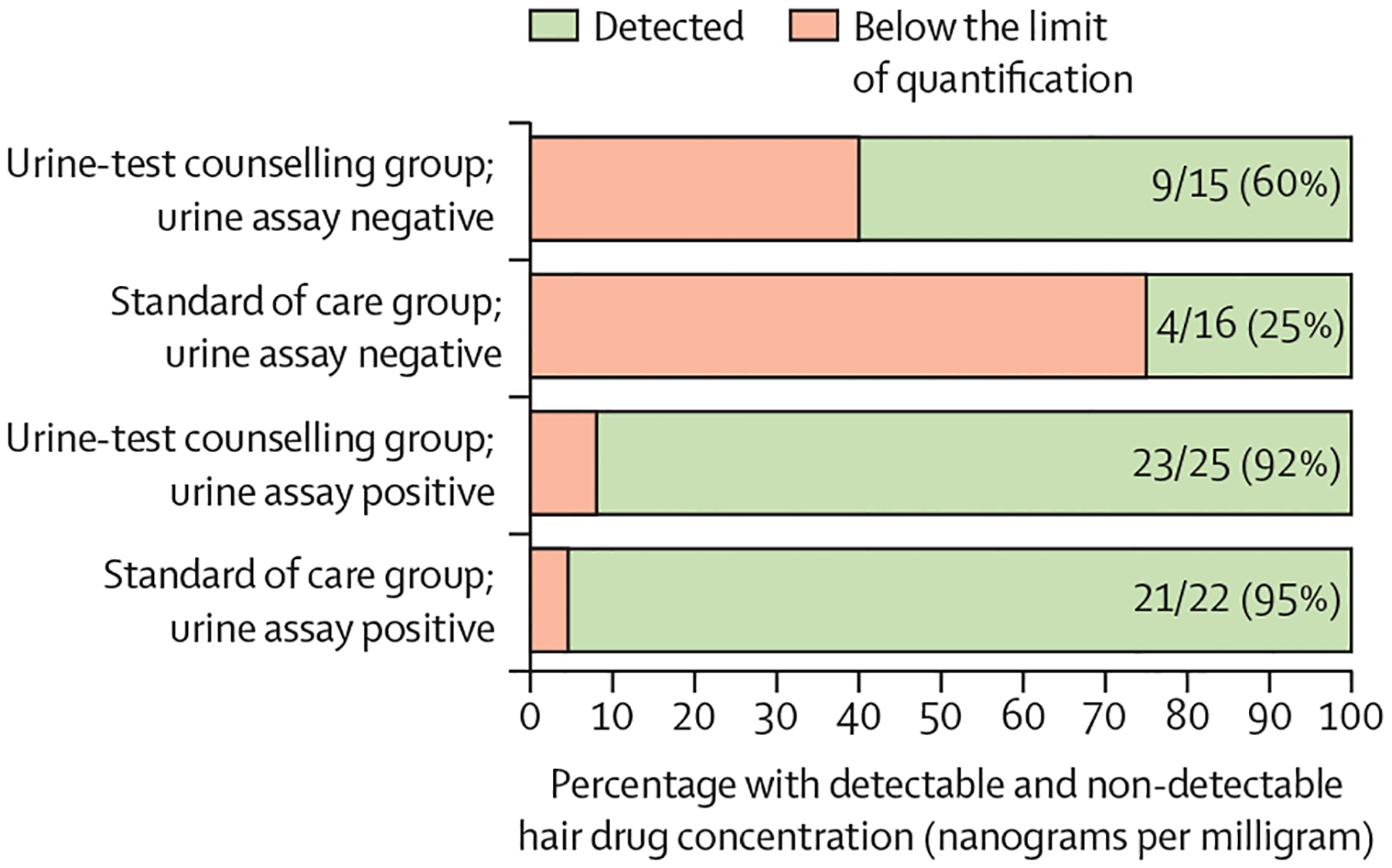
Percent with long-term non-adherence (via hair levels) in the urine-test counselling group versus the standard of care group, stratified by urine test result at month 12 When the urine assay was negative, rates of hair tenofovir level detectability were higher in the urine-test counselling group (arguing against white-coat adherence explaining these results).

Hair was collected and tested from 42 participants in the urine-test counselling group at month 12, and hair was collected from 43 participants and testing completed for 41 of those samples in the standard of care group at month 12. Nine (21%) of 42 in the urine-test counselling group had hair samples at 12 months with tenofovir concentrations below the limit of quantification compared with 15 (37%) of 41 in the standard of care group (figure 3). The relative odds of long-term non-adherence in the standard of care group compared with the urine-test counselling group were 3·53 (95% CI 1·03–12·03; p=0·044). Feedback based on the urine assay results in the urine-test counselling group seemed to decrease overall non-adherence as compared with the standard of care group, even when the urine assay was negative at the 12-month visit (percentage of hair samples with undetected tenofovir levels was 40% [6/15] in urine-test counselling group participants with a negative urine assay result *vs* 75% [12/16] among standard of care group participants with a negative urine assay result; p=0·048).

At 12 months, 56% of participants in the urine-test counselling group stated that the urine test “increased their adherence a lot”; 93% liked the urine adherence test “a lot”; and 88% would want to use the test outside of a study (table 2). No serious adverse events were reported.

**Figure 4:**
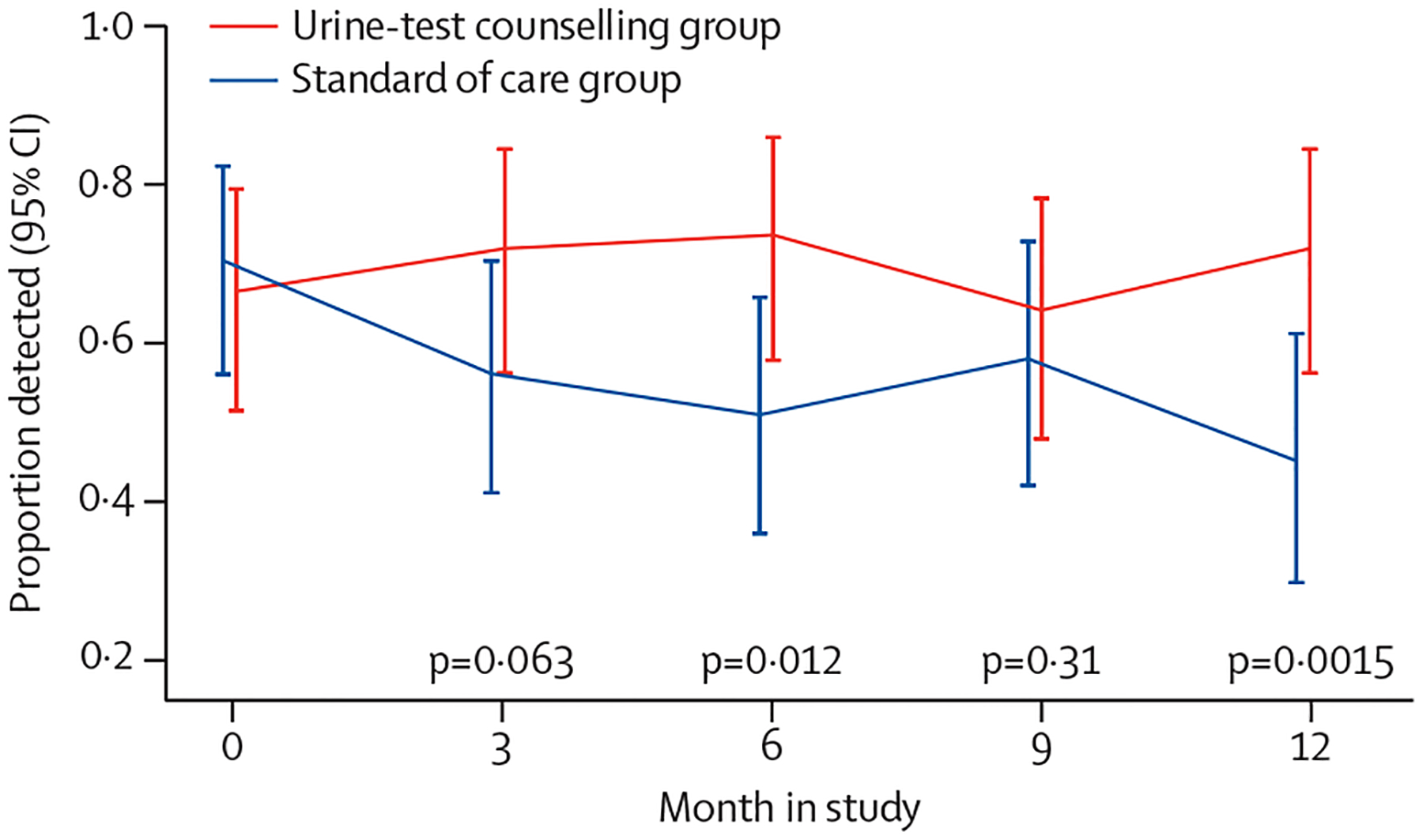
Proportion of urine tenofovir assays which were positive in each group by visit

At the baseline visit, before any intervention, the proportion of positive urine tenofovir tests was 65% (32/49) in the urine-test counselling group and 71% (36/51) in the standard of care group (figure 4). In subsequent visits, the proportion of positive urine tenofovir assays was 31 (72%) of 43 in the urine-test counselling group versus 27 (56%) of 48 in the standard of care group at 3 months; 31 (74%) of 42 versus 24 (51%) of 47 at 6 months; 27 (64%) of 42 versus 25 (58%) of 43 at 9 months; and 31 (72%) of 43 versus 19 (45%) of 42 at 12 months (figure 4).

## Discussion

Our group developed and validated one of the first point-of-care urine-based assays to assess adherence to tenofovir-based antiretroviral therapy and PrEP via an immunoassay.^13,16^ For the first time, this study piloted the ability of the urine tenofovir assay to increase long-term adherence to PrEP, specifically among women in Kenya who were not in serodiscordant couples. Real-time feedback on the urine tenofovir result in the urine-test counselling group both increased the prevalence of positive urine tests at the 3-month, 6-month, and 12-month visit, and decreased long-term non-adherence to PrEP as assessed by hair concentrations of tenofovir at 12 months. Moreover, as shown in other studies,^14,19^ the urine test was highly acceptable to participants. The positive effect on long-term adherence of the urine assay-informed counselling intervention argues against white-coat adherence, where adherence could improve temporarily just before study visits for reasons of social desirability.^28^ A larger randomised controlled trial to evaluate the impact of real-time biofeedback from the urine tenofovir assay on both long-term adherence and seroconversion outcomes among both men and women in sub-Saharan Africa is being planned.

Drug-level feedback with subsequent counselling increases adherence in other chronic diseases states, such as diabetes or hypertension.^25,29,30^ The urine tenofovir immunoassay has an advantage over other drug concentration assays in being able to provide feedback in real time, which can allow for supportive messaging (appendix p 3), motivational interviewing, or the application of other adherence interventions at the clinic or study visit. Implementation studies in the future using this test should adopt non-judgemental, supportive counselling recommended by the women surveyed at KEMRI.^19^ Other assays for PrEP monitoring which use LC-MS/MS, such as tenofovir assays in plasma, dried blood spots, or hair, take some time to process, leading to delayed feedback to participants. For example, two studies in sub-Saharan Africa evaluated the effect of adherence feedback with tenofovir-diphosphate concentrations from dried blood spots samples among adolescent girls and young women.^9–11^ These studies were informed by qualitative data from the VOICE PrEP efficacy trial with women in sub-Saharan Africa, which found that PrEP participants desired feedback about their PrEP adherence with pharmacological testing and tailored counselling.^7^ The HPTN 082 trial randomly assigned Zimbabwean and South African young women to standard adherence support (counselling, two-way SMS text, and adherence clubs) with or without feedback based on tenofovir-diphosphate concentrations in dried blood spots. There was no difference in adherence at 6 months by group.^9^ The PrEP SMART study with South African young women also tested the effect of feedback counselling based on tenofovir-diphosphate concentrations from dried blood spots but this study similarly did not find a statistically significant effect of drug concentration feedback counselling on PrEP adherence (as assessed by objective long-term metrics).^11^ In both studies, the delay in receiving feedback from the PrEP adherence results in dried blood spots was 4 weeks or longer. In a qualitative study, PrEP SMART participants commented on the long delay in receiving their results back from the laboratory as a barrier to intervention effectiveness.^31^ Our point-of-care urine test allows for immediate feedback, which might have led to its success in this trial in improving adherence as assessed by long-term metrics.

Women not in serodiscordant couples often have low rates of adherence^1,2^ which is why we engaged with this population in this pilot trial. The increased test positivity in urine and the lower rates of long-term non-adherence in the intervention group in this study are promising and argue against just a short-term increase in adherence (ie, white-coat adherence).^28^ Even when the urine tenofovir assay was negative, rates of detectability of tenofovir in hair were higher in the urine-test counselling group than the standard of care group. This could indicate that just knowing that urine tests were being monitored for tenofovir led to increased pill-taking.^28^ Moreover, in a before-and-after intervention trial for those living with HIV on antiretroviral therapy, counselling informed by the urine tenofovir assay significantly increased virological suppression rates.^14^ The initial success of the PUMA study summarised here and the study among people with HIV in Namibia^32^ paves the way for larger randomised clinical trials to study the urine tenofovir assay in sub-Saharan Africa, comparing urine assay-informed counselling to standard-of-care counselling in the context of PrEP and antiretroviral therapy. The urine tenofovir assay is inexpensive to produce and is expected to be priced at approximately US$1 per test. Potential cost savings of using the urine point-of-care tenofovir assay in adherence counselling were seen in the pilot study in Namibia, where the addition of urine tenofovir testing to the standard of care was estimated to save US$1071 per virologically unsuppressed patient.^33^ The urine tenofovir test is expected to be cost-effective as an adherence intervention for PrEP given that current methods to provide feedback on drug concentrations are much more expensive, as are other resource-intensive interventions.^6^

There are several limitations to this study, notably its small sample size. However, despite the size of the study, power calculations showed we could show effectiveness, which we did: urine tests were significantly more likely to be positive in the intervention group, and hair levels more likely to be negative in the control group. A larger randomised study with implementation outcomes is being planned on the basis of these promising results. The higher proportion of positive urine tests in the intervention group could be a result of white-coat adherence but the hair tenofovir concentration data were not consistent with white-coat pattern dosing, but long-term impacts on adherence of the urine testing and feedback. Deviations from the protocol occurred as a result of the COVID-19 pandemic and associated lockdowns: specifically, dried blood spots were not collected for additional adherence monitoring and hair was only collected at 12 months. Missing data might affect the validity of results, although we had urine assays from all participants and few missing hair tenofovir values. Finally, this study cannot be generalised to other populations on PrEP such as men; women not in serodiscordant couples have historically had lower rates of adherence to PrEP than other groups.

In conclusion, we have demonstrated for the first time that an intervention incorporating a urine assay-based tenofovir adherence test into PrEP counselling increased both short-term and long-term metrics of adherence. Given the urine test’s low cost, the urine test could cost-effectively enhance the efficacy of PrEP, particularly among populations with significant adherence challenges. The results from this pilot trial (and from the study in the context of HIV treatment)^14^ support the need for large, randomised controlled trials testing the urine tenofovir assay-based intervention versus standard of care adherence counselling in diverse settings to potentially support adoption of the urine test as part of PrEP and antiretroviral therapy management worldwide.

## Supplementary Material

Supplementary material

## Figures and Tables

**Table 1:** Baseline characteristics

	Urine assay counselling group (n=49)	Standard of care group (n=51)
**Characteristics**		
Age at enrolment (years)	33·3 (10·6)	33·8 (8·2)
Years of education	10·2 (2·9)	9·2 (2·8)
Monthly income in past 3 months (KES)	6408 (7680)	9002 (9955)
Age at sexual debut (years)	17·3 (2·9)	17·4 (2·8)
Days between PrEP initiation and study enrolment	85·8 (7·6)	84·2 (5·8)
Took PrEP every day in past month	41 (84%)	39 (77%)
PrEP adherence (via visual analogue scale) in past month among those who did not take PrEP every day	62·1 (35·5)	74·9 (19·3)
Employment status (past 3 months)*		
Labourer or semiskilled	20 (41%)	13 (25%)
Trade or sales	14 (29%)	22 (43%)
Student	2 (4%)	0
Professional	2 (4%)	3 (6%)
Farming or animal raising	4 (8%)	6 (12%)
Housewife	6 (12%)	6 (12%)
Multiple	1 (2%)	1 (2%)
Primary source of household income (past year)		
Own work	30 (61%)	32 (63%)
Husband’s work	7 (14%)	6 (12%)
Family	2 (4%)	4 (8%)
Other	10 (20%)	9 (18%)
HIV status of primary or main sexual partner		
Negative	12 (25%)	12 (24%)
Unknown	32 (65%)	33 (65%)
No primary or main partner	5 (10%)	6 (12%)
Married	19 (39%)	20 (39%)
On birth control	36 (74%)	34 (67%)
Pregnant	2 (4%)	2 (4%)
Travel time to clinic		
30–59 min	9 (18%)	7 (14%)
1–2 h	16 (33%)	24 (47%)
>2 h	24 (49%)	20 (39%)
**Sex behaviour in past month**		
Number of sex partners	1 (1–3)	1 (1–3)
Instances of sex	8 (3–13)	8 (3–18)
At least one instance of condomless sex	38 (78%)	40 (78%)
At least one new sex partner	19 (39%)	17 (33%)
At least one HIV-positive sex partner	14 (29%)	15 (29%)
At least one sex partner of unknown HIV status	11 (22%)	11 (22%)
At least one HIV-negative sex partner	11 (22%)	14 (28%)
Exchanged sex for money or a gift	22 (45%)	20 (39%)

Data are mean (SD), n (%), or median (IQR). PrEP=pre-exposure prophylaxis.

* Multiple responses allowed.

**Table 2:** Acceptability of point-of-care urine tests among the urine assay counselling group participants

	Baseline (n=49)	Month 6 (n=42)	Month 12 (n=43)
**How did knowing the PUMA result during the study visit impact your PrEP adherence after your visit?**			
It increased my adherence a lot	··	27 (64%)	24 (56%)
It increased my adherence a little	··	5 (12%)	5 (12%)
It did not impact my adherence at all	··	9 (21%)	13 (30%)
It decreased my adherence a little	··	0	0
It decreased my adherence a lot	··	0	0
Missing response	··	1 (2%)	1 (2%)
**How much did you like or dislike receiving PrEP adherence results in real time?**			
Liked very much	45 (92%)	40 (95%)	40 (93%)
Liked a little	3 (6%)	1 (2%)	1 (2%)
Disliked a little	0	0	1 (2%)
Missing response	1 (2%)	1 (2%)	1 (2%)
**If not part of a study, how likely would you be to want to know PrEP adherence results using PUMA?**			
Very likely	42 (86%)	36 (86%)	38 (88%)
Somewhat likely	2 (4%)	2 (5%)	2 (5%)
Neutral	2 (4%)	2 (5%)	0
Somewhat unlikely	1 (2%)	0	1 (2%)
Very unlikely	1 (2%)	1 (2%)	1 (2%)
Missing response	1 (2%)	1 (2%)	1 (2%)

Data are n (%). PrEP=pre-exposure prophylaxis. Point-of-Care Urine Monitoring for Adherence study.

## Data Availability

De-identified participant data and a data dictionary defining each field in the dataset will be made available to investigators upon request to the corresponding author after publication and sent securely without restrictions. The study protocol, statistical analysis plan, and informed consent are also available upon request.

## Research in context

### Evidence before this study

Adherence to pre-exposure prophylaxis (PrEP) is often assessed by use of pharmacological metrics, where drug concentrations are measured in a biomatrix such as dried blood spots, hair, plasma, or urine, given the limitations of self-reported adherence. Methods for objective adherence monitoring to date, however, take time and expense to process. A newly developed immunoassay using an antibody directed against tenofovir (the agent used in oral PrEP) detects recent pill-taking in urine at the point of care. A point-of-care assay can allow for immediate adherence assessment and intervention in real time. PrEP counselling is standardised in Kenya and other sub-Saharan Africa countries and performed quarterly at each PrEP visit. This is the first description of an interventional study to inform counselling with results from the urine tenofovir assay, specifically among women in non-serodiscordant couples in Kenya (where the male partner is not known to be living with HIV), a population with historically low PrEP adherence. No previously reported interventional study using a urine tenofovir assay in the setting of PrEP has been found in the literature after searching PubMed, conference abstracts from the Conference on Retroviruses and Opportunistic Infections, International AIDS Society, the HIV Research for Prevention Conference, and the Adherence International Association of Providers of AIDS Care meeting. Search terms used were “urine assay”, “tenofovir”, “point-of-care”, “lateral flow assay”, “pre-exposure prophylaxis”, “PrEP”, “adherence”, “intervention”, “tenofovir disoproxil fumarate/emtricitabine”, and “adherence counselling”. Searchs were restricted to articles published in English from Jan 1, 2015 to March 1, 2024. Oral PrEP with tenofovir disoproxil fumarate plus emtricitabine is being expanded worldwide for prevention of HIV acquisition and is provided without cost to users in many countries in sub-Saharan Africa, including Kenya. However, adherence to oral PrEP can be low, especially among sexually active women without serodiscordant partners (where the partner is not known to be living with HIV). Recent systematic reviews have shown rates of PrEP discontinuation and rates of non-adherence to PrEP are both high, especially when adherence interventions aren’t applied.

### Added value of this study

This study examined, for the first time, an adherence intervention employing the urine tenofovir assay for women on PrEP in Kenya in non-serodiscordant partnerships in a pilot trial. At 12 months, the proportion of urine tests positive for tenofovir was significantly higher in the intervention group, which employed urine tenofovir assay-informed counselling, compared with standard-of-care counselling. Relative odds of long-term non-adherence to PrEP were higher in the standard-of-care group than in the intervention group. Increase in both short-term and long-term adherence metrics and the fact that long-term adherence was better in the intervention group at 12 months, even when the urine assay was negative at that visit, argues against the idea that women were employing white-coat adherence (ie, temporarily increasing adherence before a visit).

### Implications of all the available evidence

Although PrEP has the potential to help end the HIV epidemic along with HIV treatment, adherence to PrEP can be low. Adherence interventions using drug-level feedback have been limited to date by a lack of a real-time metric since drug concentrations analysed by use of liquid chromatography tandem mass spectrometry take time and laboratory expertise to process. Simple, low-cost interventions to increase PrEP adherence are urgently needed. The urine tenofovir assay is inexpensive, widely scalable, and can provide information on recent pill-taking in real time. Given the promise of PrEP, this point-of-care urine tenofovir test has potential to help increase adherence. The results from this pilot trial support the need for large, randomised controlled trials testing the urine tenofovir assay-based intervention versus standard of care adherence counselling in diverse settings.
