# Supplementary material for: Impact of a point-of-care urine tenofovir assay on adherence to HIV pre-exposure prophylaxis among women in Kenya: a randomised pilot trial
Source: Lancet HIV. Author manuscript; Available in PMC 2024 Sep 5. (PMC11376217; doi:10.1016/S2352-3018(24)00125-5)
Supplement: Supplementary material [file NIHMS2018078-supplement-Supplementary_material.pdf]

THE LANCET
HIV

Supplementary appendix

This appendix formed part of the original submission and has been peer reviewed. We post it as supplied by the authors.

Supplement to: Gandhi M, Glidden DV, Chakravarty D, et al. Impact of a point-of-care urine tenofovir assay on adherence to HIV pre-exposure prophylaxis among women in Kenya: a randomised pilot trial. *Lancet HIV* 2024; published online July 5. https://doi.org/10.1016/S2352-3018(24)00125-5.

Table of Contents

Figure (page 2): Picture of urine tenofovir test used in the study 2
Figure (page 3): Examples of counseling messages provided to intervention arm participants based on urine tes results...3

Figure 3: Picture of urine tenofovir test used in the study

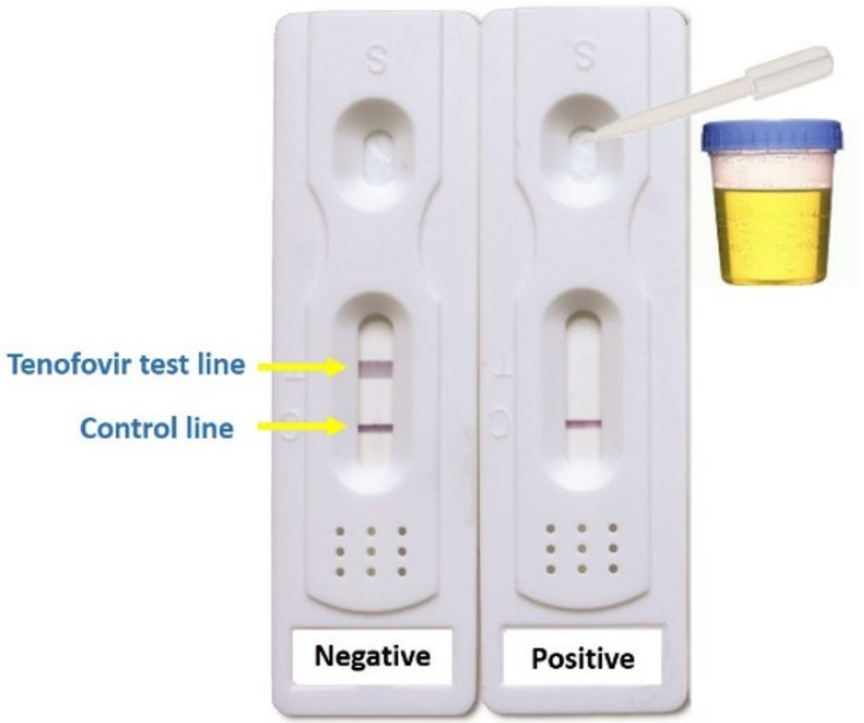

Figure 4: Example of counseling messages provided to intervention arm participants in the PUMA trial

**Urine TFV test POSITIVE**	**Key message:** You are doing really well! You are taking the PrEP. Keep up the good work and remember that taking one PrEP pill every day is needed for strong protection against HIV.
**Urine TFV test NEGATIVE**	**Key message:** It looks like you haven't been able to take the PrEP pills, at least recently. Are you still interested in PrEP? If yes, how can we help you take the medication? Talk to us about what makes it hard to take and let us see how we can help.
